# Paragraph 138

**Published:** 1887-03

**Authors:** Ad. Lippe

**Affiliations:** Philadelphia


### PARAGRAPH 138.

Ad. Lippe, M. D., Philadelphia.

Paragraph 138 of Samuel Hahnemann's *Organon of Homœopathic Medicine* reads as follows: "Provided all the conditions before stated (§§ 124–127) (which are necessary to the trial of a pure experiment) be complied with, the symptoms, modifications, and changes of the health that are visible during the action of the medicine depend upon that substance alone, and ought to be noted down as properly belonging to it, even if similar symptoms, occurring spontaneously, should have been experienced a *long time before* by the person on whom the experiment is made. The re-appearance of those symptoms, in the course of the experiment, only proves that in virtue of his own constitution this person has a special tendency to admit of their manifestation. In this case they are the effects of the medicine, for it cannot be said that they came of themselves at a moment when a powerful medicinal agent exercised its sway over the entire organism."

In the *North American Journal of Homœopathy*, November, 1886, on page 760, we find a report of a meeting of the Society for Medico-Scientific Investigation, held October 12th, 1886. The object of the meeting was the rebuilding of the Temple of Therapeia. The suggestions to accomplish that purpose were made by Dr. T. F. Allen. It had, up to the 12th of October,

1886, been the belief that Samuel Hahnemann had laid the foundation of the Temple of Therapeia, that among his followers there were men like the late Constantine Hering, the Vienna provers, and others, who added their mite to the further construction of the temple. On the 12th of October, 1886, Dr. T. F. Allen suggests the rebuilding of the Temple of Therapeia. The suggestion to "*rebuild*" implies that the structure of the temple has proved itself a failure. The onus of the proof that the Temple of Therapeia, erected by Hahnemann, *is* a failure and requires to be laid low to be rebuilt, lies with the proponents, and their spokesman is ready to bring in his evidence. The utter absurdity of this attempted evidence will be shown presently. Dr. T. F. Allen pleads: "Many errors had crept into our work of *Materia Medica*, owing in large part to Section 138 in the *Organon*." Then follows an inaccurate quotation of Section 138. (*Hahnemann* says similar symptoms occurring spontaneously; Allen says, similar *and* spontaneous sensations.) We have given the whole section at the beginning of this paper. Men of letters, anxious to distinguish themselves, have tried in vain to trip up the great poet, Shakespeare, in error; these men have all been extinguished. The same fate awaits every medical man who will attempt to distinguish himself by charging Dr. Samuel Hahnemann with uttering erroneous statements. Paragraph 138 of the *Organon* is not an error, and its correctness is self-evident. If the organism is affected by any sickmaking power, be it a drug or miasm, or any other dynamic influence, material or immaterial, that organism, holding in suspension, in virtue of its own constitution, some special tendency, will show this latent constitutional condition during the development of the sickmaking power now invading it, and symptoms he has complained of at times long ago will appear again and leave again without medication. To illustrate the correctness of this proposition, we will just here relate a case in point. On the 12th of September, 1885, a lady consulted me. She had had for months in succession attacks of an ulcerated sore throat, which remained not only uncured, but became worse all the time. It was a clear case for Lachesis, and she took a dose of it once in ten days till December, when the throat was cured. I did not see her till October, 1886. Throat had remained well; so was the lady; had grown stout, but complained now especially of severe rheumatic pain in the *left* shoulder, morning headaches, etc. It was again a clear case for Lachesis, and she received *one dose* of the highest potency. She called a week later—all well, *but* three days after taking the Lachesis her throat became sore and remained so for nearly two days, as she expressed it, similarly but not as severely sore as formerly; it did not amount to much, she remarked; she has been well since. This case, like other experiences, confirms the correctness of every word of Section 138, and proves it tenable by positive evidence. The Homœopathic Healing Art gained a standing by the successes shown by the early pioneers, who relied solely on Hahnemann's *Materia Medica Pura* and his *Chronic Diseases*. *If* that *Materia Medica* had been "unreliable," what about the successes of those who relied on it? But Dr. T. F. Allen suggests "rebuilding." Not satisfied with charging Section 138 with being the cause of many errors, which Dr. Allen says and merely asserts have crept into our works of *Materia Medica*, trying to discredit Hahnemann, he now also reaches for C. Hering, and wants to give him a home thrust when he says, "Such works as Hering's *Guiding Symptoms*, while valuable from a clinical standpoint, were not either scientific or homœopathic." What sort of logic have we here? If the *Guiding Symptoms* are valuable from a clinical standpoint, then their clinical value must surely confirm their scientific merits; if they were not either scientific or homœopathic, how came they to be valuable from a clinical standpoint? Does not the true test of a *professedly* scientific work consist in its usefulness clinically? If it is not useful and valuable from a clinical standpoint, it may still be admired as "*scientific*" by such medical men as have not yet learned that the science of medicine consists in the knowledge how to heal the sick. As Dr. T. F. Allen admits that Hering's *Guiding Symptoms are* valuable from a clinical standpoint, it would behoove him to say also that said work is highly scientific and homœopathic.

We are told to tear down and rebuild upon the principle enunciated in Hahnemann's first edition. We are not told at what time of the day the meeting of the Society for Medico-Scientific Investigation was held, but from this last, utterly backward progressing sentence we take it for granted that the meeting was held "after dinner." Our notorious weakness for putting the most charitable construction on the shortcomings of erring brethren urges us to come to that conclusion. Still, there is a method in all this rambling talk. Dr. Richard Hughes & Company, the authors of the new *Drug Pathogenesy* by Dr. Hughes termed the *Materia Medica* of the Future, have utterly ignored Section 128 of the *Organon*. After all the biddings of T. F. Allen are complied with, after Hughes & Company have set aside Section 128 and Allen Section 138 of the *Organon*, after the *Organon* has been condemned as full of errors—errors come to light after Hahnemann's *Materia Medica* of 1830 has established "Our Healing Art"—there will be very little left of a materia medica, and the new Temple of Therapeia will be "a molehill" only, and superseding the *Drug Pathogenesy* will have to wait till Hughes' opus, the *Materia Medica* of the Future, *has come.* After that future will come the molehill temple worship—of the tail of that Kilkenny Scientific Cat.

